# Identifying the functions and biomarkers of *Codonopsis pilosula* and *Astragalus membranaceus* aqueous extracts in hepatic cells

**DOI:** 10.1186/s13020-019-0233-1

**Published:** 2019-03-20

**Authors:** Pin-Hao Ko, Chiung-Wei Huang, Hen-Hong Chang, Eric Y. Chuang, Mong-Hsun Tsai, Liang-Chuan Lai

**Affiliations:** 1 Graduate Institute of Physiology, College of Medicine, National Taiwan University, Taipei, 100 Taiwan; 2 Department of Physiology, College of Medicine, Kaohsiung Medical University, Kaohsiung, 807 Taiwan; 3 School of Post-Baccalaureate Chinese Medicine, China Medical University, Taichung, 404 Taiwan; 4 Department of Chinese Medicine, China Medical University Hospital, Taichung, 404 Taiwan; 5 Graduate Institute of Biomedical Electronics and Bioinformatics, National Taiwan University, Taipei, 106 Taiwan; 6 Bioinformatics and Biostatistics Core, Center of Genomic Medicine, National Taiwan University, Taipei, 100 Taiwan; 7 Institute of Biotechnology, National Taiwan University, Taipei, 106 Taiwan

**Keywords:** Biomarkers, *Codonopsis pilosula*, *Astragalus membranaceus*, *Citrus reticulate*, HepG2

## Abstract

**Background:**

Homeostasis is a crucial concept used to describe the condition of patients and the roles of herbs in traditional Chinese medicine. Qi-deficiency pattern is one of the conditions when loss of homeostasis and is usually characterized by symptoms including lassitude, spontaneous sweating, and a weak pulse, which are not easy to quantitate. *Codonopsis pilosula* and *Astragalus membranaceus* were usually prescribed for carriers with hepatitis and patients with metastatic colon cancer, because these patients tended to experience fatigue. However, crude drugs were prescribed based on the exterior symptoms of patients without controlling clinical setting, such as gender, age, and dietary habits. Limited molecular evidence of using gene expression as the guide for description is available. Therefore, the purpose of this study was to identify potential and objective biomarkers of these two qi-related drugs in a simplified cellular system.

**Methods:**

Aqueous extracts of crude qi-tonifying herbs, *C. pilosula* and *A. membranaceus*, and that of a qi-consuming drug, *Citrus reticulata*, were prepared. Human liver cancer HepG2 cells were treated with the extracts of qi-tonifying herbs for 24 h. Differentially expressed genes were identified using microarrays and quantitative RT-PCR (qRT-PCR) and validated in two other hepatocellular cell lines, Huh7 and L-02.

**Results:**

A total of 67 differentially expressed probes that responded to both herbs were identified. A pathway analysis revealed that these genes were involved in the development, growth, movement, and viability of the liver cells.

**Conclusions:**

After qRT-PCR validation and examination of clinical data from public domains, our results showed that two genes, *GDF15* and *HMOX1*, could serve as biomarkers in liver cells for identifying responses after treatment with *C. pilosula* and *A. membranaceus*.

**Electronic supplementary material:**

The online version of this article (10.1186/s13020-019-0233-1) contains supplementary material, which is available to authorized users.

## Background

Aqueous extract decoction is a widely used form of drug preparation in traditional Chinese medicine (TCM), and physical symptoms in patients are a crucial basis for prescribing herbal decoctions. Qi-deficiency symptoms include shortness of breath, lassitude, listlessness, spontaneous sweating, a pale tongue, and a weak pulse [1]. Many related single or complex formulas have been widely prescribed in TCM to ameliorate these symptoms [2, 3].

Qi-related crude drugs can be classified as qi-tonifying and qi-consuming drugs. The use of dried roots of *Codonopsis pilosula* and *Astragalus membranaceus*, qi-tonifying crude drugs, has been well documented in the ancient Chinese literatures, including in Ben cao gang mu [4], a few 100 years ago. *C. pilosula* is usually combined with other herbs to alleviate qi deficiency. For example, an animal study demonstrated that *C. pilosula* polysaccharide attenuated sepsis in a mouse model of cecal ligation [5]. In a randomized controlled trial, TCM formulas containing *C. pilosula* were used in patients with chronic obstructive pulmonary disease [6]. Extracts of *A. membranaceus* have been reported to accelerate wound healing, exhibit antitumor activity [7], inhibit inflammation [8], and inhibit melanogenesis through ERK pathway [9]. Because these two drugs, especially *A. membranaceus*, the most potent qi-tonifying drugs [10], were prescribed frequently in patients with liver cancer who tended to experience fatigue, they were selected for the present study. *Citrus reticulata* is a qi-consuming plant used in TCM [4]. The peel of *C. reticulata* is the major constituent of the drug. It contains many polyphenols [11], and its extract possesses antioxidant, anticancer, and antibacterial properties [12, 13].

A single drug, *C. pilosula* or *A. membranaceus*, was usually prescribed on the basis of patients’ external symptoms alone with little molecular-marker evidence. Furthermore, although studies have suggested some molecular pathways might be involved in the mechanisms underlying these drugs [5, 7, 8], the overall mechanisms remain elusive. Therefore, the purpose of this study was identify potential biomarkers for qi-related drug treatment and establish the possible underlying mechanisms through biological approaches.

We hypothesized that the gene expression levels could serve as biomarkers to assess the status of cell conditions. In addition, because patients with related symptoms usually exhibit large variations in biochemical examination, hepatocellular cell lines, HepG2, Huh7, and L-02, were treated with the herbs in this study. Therefore, to identify biological functions, a genome-wide approach, microarray technique, was first used to screen differentially expressed genes and their functions. Furthermore, PCR results and clinical data from an independent cohort revealed that the expression levels of *GDF15* and *HMOX1* could serve as biomarkers for identifying responses of these botanical drugs in liver cells.

## Materials and methods

The Minimum Standards of Reporting Checklist contains details of the experimental design, and statistics, and resources used in this study (Additional file 1).

### Sample preparation

Dried roots of *C. pilosula* and *A. membranaceus* were used as crude drugs, as reported in our previous papers [14–16]. The roots were soaked in water at a ratio of 1:3 (g:mL) and boiled at 100 °C for 4 h. The decoctions were dehydrated using a freeze dryer (Panchum FD DC-3000, Panchum, Kaohsiung, Taiwan), filtered using a 0.22-µm filter (Merck Millipore, Tullagreen, Carrigtwohill, IRL), and re-dissolved in ddH_2_O.

### Quality control of crude drugs using high-performance liquid chromatography (HPLC) analysis and internal transcribed space 1 (ITS1) analysis

The details of the preparation steps and high-performance liquid chromatography (HPLC) equipment and results have been described previously [14]. Because the same herbs were used in the present study, we only showed the following key settings: The powdered roots of both plants (0.2 g) were suspended in 70% methanol and filtered through a 0.2-μm HPLC membrane. The HPLC conditions were as follows: gradient range, 10–90% methanol; flow time, 120 min; detector, 254 nm; flow rate, 1 mL/min; injection volume, 20 μL; and internal control, evodiamine.

The internal transcribed spacer (ITS) analysis protocols have been described previously but only ITS2 sequencing results have been published [14, 15]. The ITS1 for all herbs were sequenced using the following primers: (forward) 5′-GGAAGTAAAAGTCGTAACAAGG-3′; (reverse) 5′-TCCTCCTCCGCTTATTGATATGC-3′ (Table 1). The botanical origins of the plants were validated by comparing the ITS sequences with those in the National Center for Biotechnology Information (NCBI) nucleotide database.

**Table 1:** Internal transcribed spacer 1 (ITS1) sequences of the three crude drugs

*Codonopsis pilosula*
GTCGAAACCTGCACAGCAGAACGACCCGCGAACACGTGAACAACACCGGGGACGCGGGCTTGCCCGTGGCCCCTTGCCGTCGGCGCATGCACCCGCCCAACCACTTGGTGGAAGGGAGCATGCGTGCGTCGTTCGGCGCCAAACGAACCCCGGCGCGATCCGCGCCAAGGAAAACTTAACTCAAAGAGCGCCACGTCCTCCCGTCGCCCCGTTCGCGGTGTGCGCACGGTTGGGTGGTCGCTTCTTAGTGAAAAACACAAACGAC
*Astragalus membranaceus*
GGGGGGTGTTTTGCACCACGACCTCCCTTTGGGTGGGGTGTGGTGCGCAATGCGTTCCCCCTCCTGCCCGAACACAAACCCCGGCGCTCAATGCGCCAAGGAACTAAAATTCGATCAATGTGCCCCGTCGGCCCGGAGACGGTGCTTCGGCGGTGGTGCCTTGTCACATGATACAGAATGACTC
*Citrus reticulate*
GAACCTGCCCAGCAGAACGACCCGCGAACCAGTTGATATCACCGGCGGCGGGAGGGGGTGCGCRTCCGCAACGGGCGCTCCTCCTTCCCGCCCCATGCCGCGGGGAGAGGGACTCGTCCCGCTCCCGGCTGGCGAAACAACRAACCCCCGGCGCGGACTGCGCCAAGGAAATCTAACGAGAGAGCACGCTCCCGCGGCCCCGGAGACGGTGCGCCGCGGGGTGCGGCGCCTTCTTTCACATGTATCCAAAAC

### Cell culture

The cancerous liver cell lines HepG2 and Huh7 cells were cultured in Dulbecco modified Eagle medium (GIBCO, Carlsbad, CA, USA) containing 1% streptomycin/puromycin (Biological Industries, Kibbutz Beit-Haemek, Israel) and 10% fetal bovine serum (Biological Industries). The human fetal hepatocyte line, L-02, a kind gift from Dr. Hui-Lin Wu from Hepatitis Research Center, National Taiwan University Hospital, was cultured in Roswell Park Memorial Institute medium 1640 (GIBCO). All cells were cultured at 37 °C in a humidified atmosphere containing 5% CO2.

### Cell viability assay

The cells were seeded at a density of 3000 cells per well into a 96-well microtiter plate and treated with various concentrations (0–5 mg/mL) of each drug for 24 h. Cells were incubated with 0.46 mg/mL of 3-(4,5-dimethylthiazol-2-yl)-2,5-diphenyltetrazolium bromide (MTT, BioTek, Winooski, VT, USA) at 37 °C and in an atmosphere containing 5% CO2 for 1.5 h. A fixed volume was sampled from each well and dissolved in 100 μL of dimethyl sulfoxide. Absorbance values were measured using a microtiter plate reader (BioTek, Winooski, VT, USA) at 570 nm.

The BrdU assay served as another detection method for cell viability. The BrdU proliferation analysis was performed with the BrdU cell proliferation ELISA kit according to the manufacturer’s protocol (Abcam). Cells were incubated with BrdU buffer for 3 h, and the wavelength was measured at 450 and 550 nm.

### DNA ladder assay

The HepG2 cells were seeded at a density of 1 × 10^5^ cells per well into a 6-well microtiter plate and treated with 3 mg/mL *C. pilosula* or *A. membranaceus* for 48 h. DNA was extracted according to the manufacturer’s protocol (Qiagen, Hilden, Germany). Three micrograms of DNA were loaded in 1.5% agarose gel (Seakenm, Lonza Rockland, ME, USA). DNA detection was done by safe DNA gel stain system (Invitrogen, USA) with BioSpectrum Imaging System (UVP, Upland, CA, USA). A newly synthesized compound 1-(9′-methyl-3′-carbazole)-3,4-dihydro-beta-carboline (MCDC) [17] was served as positive control.

### RNA extraction and quantitative RT-PCR

Total RNA was extracted using the TRIpure reagent (Roche Diagnostics, Branchburg, NJ, USA) according to the manufacturer’s protocol. One microgram of total RNA was reverse transcribed to cDNA by using a high-capacity cDNA reverse transcription kit (Applied Biosystems, Carlsbad, CA, USA). Ten percent of each cDNA sample was used as a template, and the mRNA levels of different genes were quantified through quantitative RT-PCR (qRT-PCR) analysis by using primers presented in Table 2. The intensity of SYBR Green was measured using StepOnePlus Real-Time PCR Systems (ThermoFisher, Waltham, MA USA) and OmicsGreen qPCR 5× Master Mix (OmicsBio, Taipei, Taiwan) according to the manufacturers’ instructions.

**Table 2:** Primer sequence for quantitative RT-PCR

Gene name	Primers (5′ → 3′)
*AKR1B15*	F-CCCTTTGACTGGCCTAAAGAG R-AATGTGGCGATATTCTGCATCA
*GCNT3*	F-GCTGCTATATGCTGCTGGC R-CCTCTTTGCTGGAAGTTTCAGG
*GDF15*	F-GACCCTCAGAGTTGCACTC R-GTGAGTATCCGGACTGCAG
*HAMP*	F-CTGACCAGTGGCTCTGTTTTC R-GATGGGGAAGTGGGTGTCTC
*HEY1*	F-GTTCGGCTCTAGGTTCCATG R-CTTCTCAAA AGCACTGGGTACC
*HMGCS1*	F-CGCTGCTATTCTGTCTACTGC R-CAGCAACATCCGAGCTAGAG
*HMOX1*	F-CAAGACTGCGTTCCTGCTCA R-GGCAGAATCTTGCACTTTGTTG
*IGFBP1*	F-TGGGACGCCATCAGTACCTA R-CTTCTCCTGATGTCTCCTGTG
*MT1E*	F-TCAGGTTGGGAGGGAACTCA R-GAAAAATGCTGTCCTGCCCC
*ACTN*	F-GGGAAATCGTGCGTGAC R-CAAGAAGGAAGGCTGGAA

F, forward; R, reverse

### Human genome microarray analysis and Ingenuity Pathway Analysis (IPA)

An Illumina TotalPrep RNA Amplification Kit (Ambion Inc, Austin, TX, USA) and the T7 Oligo (dT) reagent were used to amplify total RNA. The cRNA was mixed with an equal volume of hybridization buffer, hybridized to Illumina Human HT-12 v4 BeadChips (Illumina) arrays at 58 °C for 16 h, and stained using the dye streptavidin-Cy3. An Illumina BeadArray Reader was used to read intensities, and the results were analyzed using BeadStudio v3.1 software. Background-adjusted signals were normalized using a quantile normalization algorithm, and the Partek Genomics Suite (Partek Incorporated, St. Louis, MO, USA) was used for principal components analysis (PCA). Cellular functions of the differentially expressed genes were examined using the Ingenuity Pathway Analysis (IPA) tool (Ingenuity Systems Inc. Redwood City, USA). Results of the microarray analysis are uploaded to the Gene Expression Omnibus (GEO) database with accession numbers GSE115506.

### Cell cycle analysis

Cultured cells were harvested through trypsinization, treated with 0.5% Triton X-100 (Sigma, Saint Louis, MO, USA) in phosphate-buffered saline, and fixed with 100% methanol (Sigma). Cell pellets were then treated with 20 μg/mL propidium iodide (Life Technologies, Eugene, Oregon, USA), 0.1% Triton-X-100 (Sigma) and 100 μg/mL RNase A (Sigma) for 10 min. The suspension was analyzed using Beckman Coulter FC500 (Beckman, Brea, CA, USA) and ModFit LT analysis software.

### Protein extraction and western blotting

Cells were lysed in radioimmunoprecipitation assay lysis buffer (Sigma). A total of 25 μg of protein lysate was loaded in each lane, separated by 10% sodium dodecyl sulfate–polyacrylamide gel electrophoresis, and transferred to a polyvinylidene difluoride membrane (Bio-Rad Laboratories). We used the following primary antibodies: rabbit antihuman GCNT3, GDF15, HMOX1, and GAPDH polyclonal antibodies (GeneTex, Irvine, CA, USA). HRP-conjugated goat antirabbit IgG polyclonal antibody (GeneTex) was used as the secondary antibody. Visualization was performed using an enhanced chemiluminescence system (Millipore, Billerica, MA, USA). Images were analyzed using ImageJ software (http://rsb.info.nih.gov/ij/).

## Results

### Quality control of three crude drugs

**Fig. 1:**
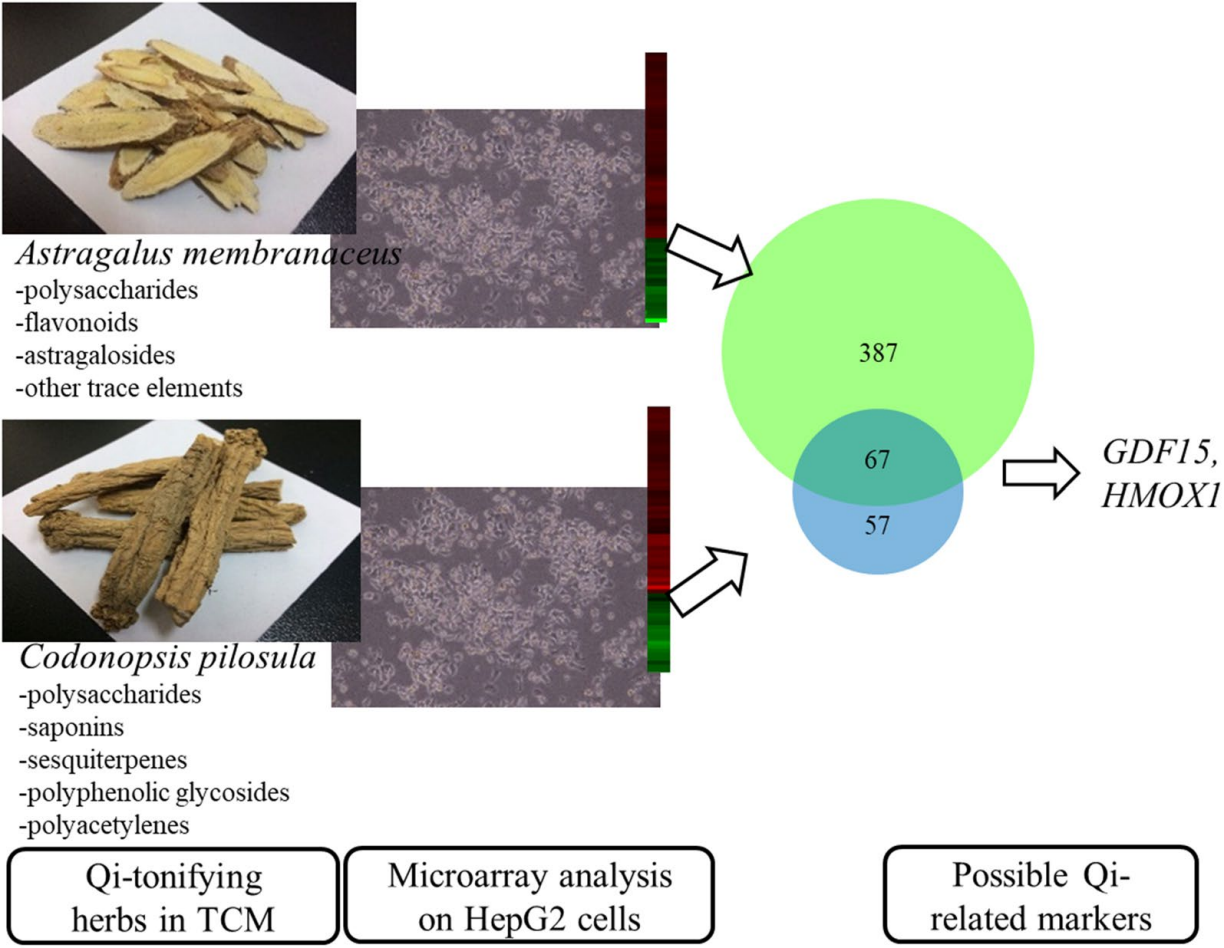
Representative photographs and work flow for identifying genes responsive to *C. pilosula* and *A. membranaceus* treatments

The workflow for identifying differentially expressed genes in HepG2 cells treated with *C. pilosula* or *A. membranaceus* was shown in Fig. 1. To examine the identity of crude drugs, high-performance liquid chromatography (HPLC) analysis and internal transcribed spacer (ITS) analysis were performed. The crude drugs used in this study are the same as that used in our previous study [14]. HPLC and ITS2 sequences data were published previously [14]. The results of HPLC confirmed the ingredients from same batch with same quality. However, ITS1 sequences have not yet been tested. Therefore, in this study, we sequenced ITS1 of *C. pilosula*, *A. membranaceus*, and *C. reticulata* (Table 1) and validated the botanical identity of these plants by comparing their sequences with those in the NCBI nucleotide database.

### Growth inhibitory effects of crude drugs in HepG2 cells

**Fig. 2:**
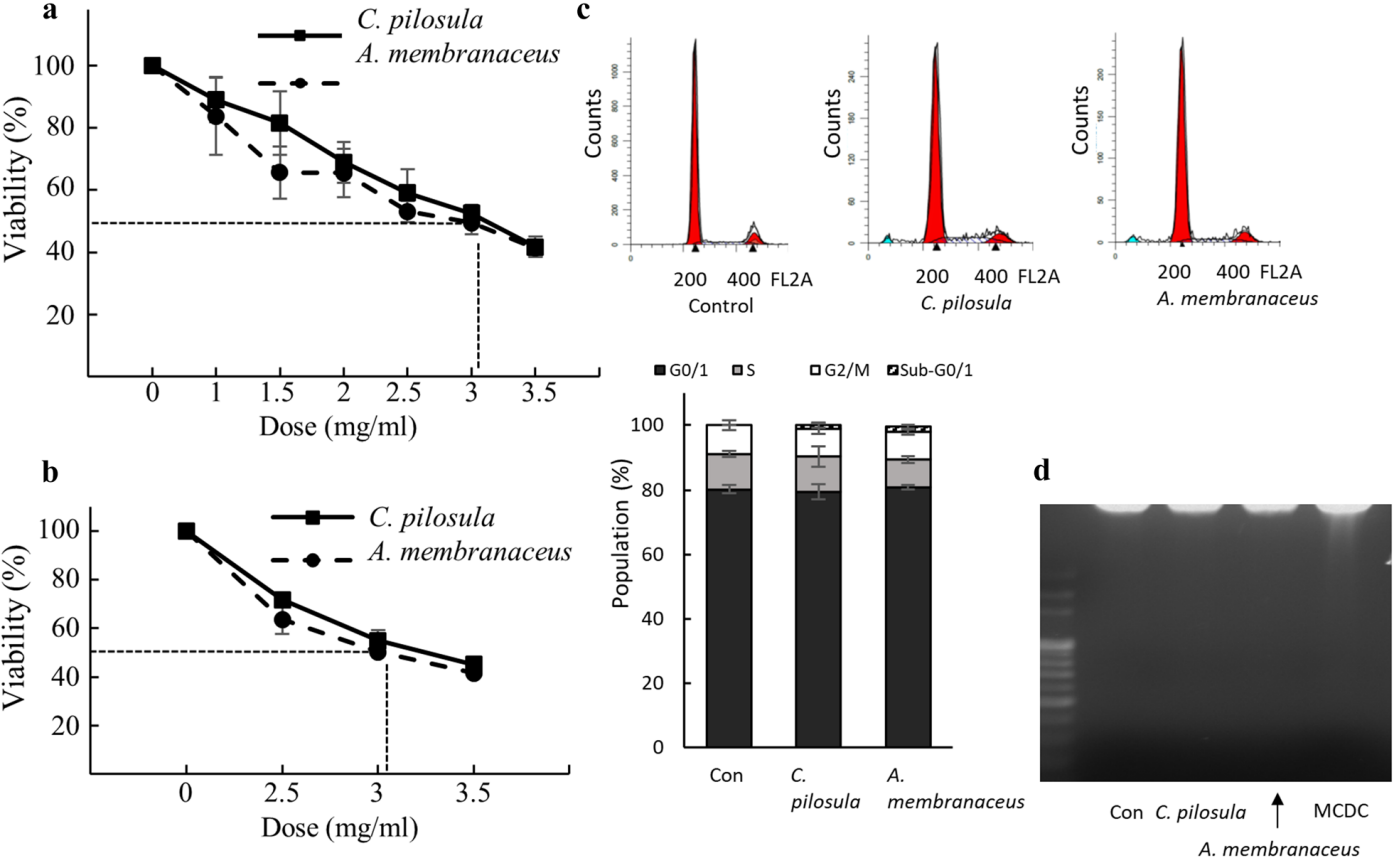
Cytotoxic effects of *C. pilosula* and *A. membranaceus* on HepG2 cells. Cytotoxic effects of crude drugs were measured using the **a** MTT and **b** BrdU assay at 48 h. **c** Cell cycle analysis. HepG2 cells were stained with propidium iodide and subjected to flow cytometry analysis after 48 h of treatment with 3 mg/mL *C. pilosula* and *A. membranaceus*. The stacked bar chart summarizes three independent cell cycle experiments. **d** DNA ladder assay. DNA from HepG2 cells was extracted after 48 h of treatment with 3 mg/mL *C. pilosula* and *A. membranaceus*. 5 μM of 1-(9′-methyl-3′-carbazole)-3,4-dihydro-beta-carboline (MCDC) was served as positive control. All experiments were repeated at least three times and results are presented as mean ± SEM

To determine the optimal conditions for testing the effects of crude drugs (*C. pilosula* and *A. membranaceus*), dose–response studies were conducted using HepG2 cells (Fig. 2). Cytotoxic effects of the crude drugs were measured using the MTT (Fig. 2a) and BrdU (Fig. 2b) assays at 48 h. The dose close to a 50% inhibition rate was 3 mg/mL (Fig. 2a, b) for both *C. pilosula* and *A. membranaceus*; this dose was used for the following experiments.

Because 3 mg/mL of *C. pilosula* or *A. membranaceus* caused nearly 50% of growth inhibitory effects on HepG2 cells, we examined whether this phenomenon was caused by the triggering of apoptosis. Approximately only 2% of cells exhibited in the sub-G0/1 peaks of the cell cycle. Only 1-(9′-methyl-3′-carbazole)-3,4-dihydro-beta-carboline (MCDC), a known compound that induced strong cytotoxic and apoptosis effect against kinds cancer cells [17], triggered DNA ladder effect on HepG2 cells but *C. pilosula* and *A. membranaceus* did not (Fig. 2d). These two botanic drugs did not induce strong apoptosis on HepG2 cells with this dosage.

### Downstream pathways of *C. pilosula* and *A. membranaceus*

**Fig. 3:**
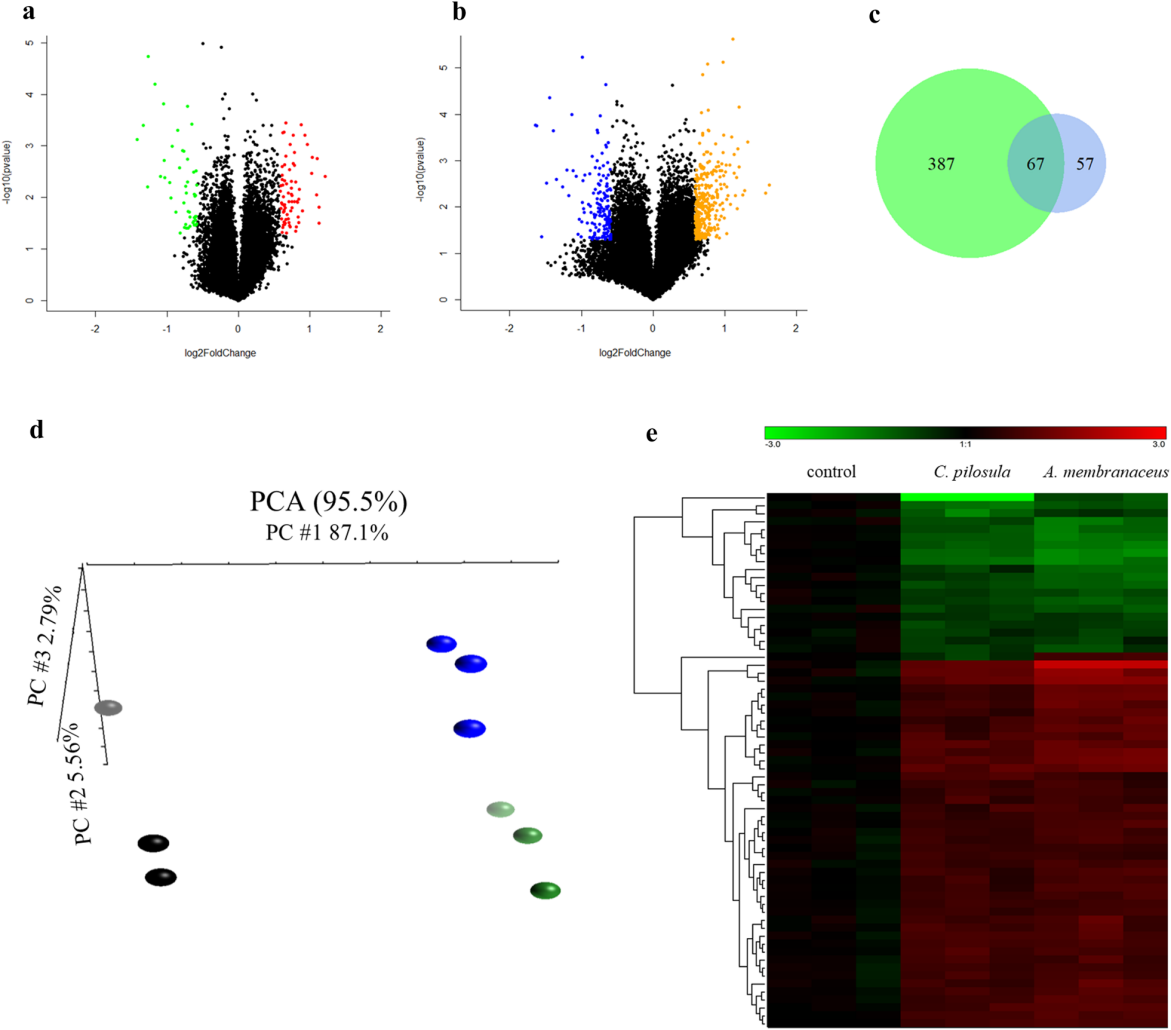
Identification of differentially expressed genes in HepG2 cells treated with *C. pilosula* and *A. membranaceus*. **a** Volcano plots of HepG2 cells treated with *C. pilosula* for 24 h at a dose of 3 mg/mL. Total RNA was extracted and genomic profiling was performed using Illumina Human HT-12 v4 BeadChips. The red spots represent probes with fold changes ≥ 1.5× and *P *< 0.05, whereas the green spots represent probes with fold changes < 1.5× and *P* < 0.05. **b** Volcano plots of HepG2 cells treated with *A. membranaceus* for 24 h at a dose of 3 mg/mL. The orange–red spots represent probes with fold changes > 1.5× and *P *< 0.05, whereas the blue spots represent probes with fold changes < 1.5× and *P* < 0.05. **c** Venn diagram of differentially expressed probes. The blue circle represents probes identified from HepG2 cells treated with *C. pilosula.* The green circle represents probes identified from cells treated with *A. membranaceus*. **d** Principal component analysis of cells treated with *C. pilosula* or *A. membranaceus* with the common differentially expressed probes (n = 67). Blue spot: *C. pilosula* treatment; green spot: *A. membranaceus* treatment; black spot: non-treated control. **e** Heatmap and hierarchical cluster analysis of differentially expressed probes responsive to both *C. pilosula* and *A. membranaceus* treatment. Upregulated and downregulated probes are denoted in red and green, respectively

Because the related pathways of these two drugs are unclear, we used microarrays to conduct the genomic profiling of differentially expressed genes in the HepG2 cells treated with two qi-tonifying plants, *C. pilosula* and *A. membranaceus*. HepG2 cells were treated with the drugs at a dosage of 3 mg/mL for 24 h, and total RNA was extracted afterwards. The selection criteria for the differentially expressed genes were a *P* value of < 0.05 and fold change of at least 1.5×. As shown in Fig. 3a and b, 124 probes were identified in the HepG2 cells treated with *C. pilosula* (Fig. 3a) and 454 probes were identified in HepG2 cells treated with *A. membranaceus* (Fig. 3b). To reduce false positives, 67 probes significantly responsive to both *C. pilosula* and *A. membranaceus* treatments were identified (Fig. 3c). To examine the drug effect at the transcriptional level, PCA was conducted using the common differentially expressed probes. As shown in Fig. 3d, the distribution of samples treated with *C. pilosula* (blue spots) and *A. membranaceus* (green spots) was closer to that of the samples treated with the control (black spots), indicating that the expression profiles of cells treated with *C. pilosula* and *A. membranaceus* were similar. The expression profiles of these differentially expressed probes were clustered and are shown in Fig. 3e.

The 67 differentially expressed probes responsive to both *C. pilosula* and *A. membranaceus* were further examined. Of these, one probe exhibited an inconsistent pattern in both treatments and was hence removed from further analysis; of the remaining 66 probes corresponding to 66 genes, 20 genes were downregulated and 46 were upregulated. To explore the disorders and functions of these qi-tonifying related genes, IPA was performed. The results of the pathway analysis revealed that these genes are involved in organismal injury, reproductive system diseases, and cancer (Table 3). The top four molecular and cellular functions controlled by these genes were related to the development, growth, movement, and viability of cells (Table 4).

**Table 3:** Top five diseases and disorders involving differentially expressed genes that responded to both *C. pilosula* and *A. membranaceus* identified using Ingenuity Pathway Analysis

Name	*P* value	Molecules
Organismal injury and abnormalities	2.42E−02 to 6.93E−07	53
Reproductive system disease	2.42E−02 to 6.93E−07	35
Cancer	2.42E−02 to 1.25E−06	46
Gastrointestinal disease	2.42E−02 to 1.25E−06	29
Hepatic system disease	2.10E−02 to 1.25E−06	18

**Table 4:** Top five molecular and cellular functions involving differentially expressed genes that responded to both *C. pilosula* and *A. membranaceus* identified using Ingenuity Pathway Analysis

Name	*P*-value	Molecules
Cellular development	2.12E−02 to 7.19E−07	22
Cellular growth and proliferation	2.12E−02 to 7.19E−07	24
Cellular movement	1.89E−02 to 9.66E−06	15
Cell death and survival	2.12E−02 to 2.41E−05	24
Amino acid metabolism	6.98E−03 to 5.50E−05	6

### Validation of probes related to qi-tonifying herbs by using quantitative RT-PCR

**Fig. 4:**
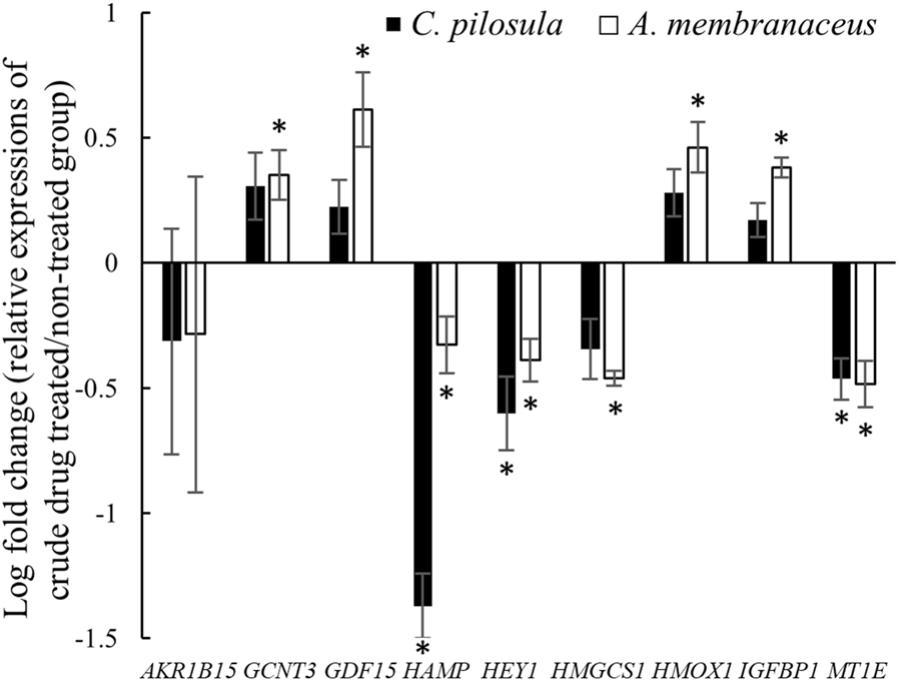
Validation of differentially expressed genes by using quantitative RT-PCR. Expression levels of nine genes were measured in HepG2 cells treated with *C. pilosula* or *A. membranaceus* (3 mg/mL) for 24 h by using qRT-PCR. Internal control: *ACTB*. The relative expression levels of genes were normalized to the non-treated group. All experiments were repeated at least three times and results are presented as mean ± SEM. *P < 0.05, Student *t* test

To validate these drug-related genes as biomarkers for qi-tonifying herbs, we selected the top nine genes with maximal changes from the common genes responsive to qi-tonifying herbs and validated their expression levels through qRT-PCR. As shown in Fig. 4, the expression levels of *GCNT3*, *GDF15*, *HMOX1*, and *IGFBP1* were significantly (*P* < 0.05) upregulated after *A. membranaceus* treatment and slightly upregulated after *C. pilosula* treatment. Conversely, the expression levels of *HAMP*, *HEY1*, *HMGCS1*, and *MT1E* were significantly (*P* < 0.05) downregulated after both *A. membranaceus* and *C. pilosula* treatment.

### Effects of qi-consuming crude on drug-related gene markers

**Fig. 5:**
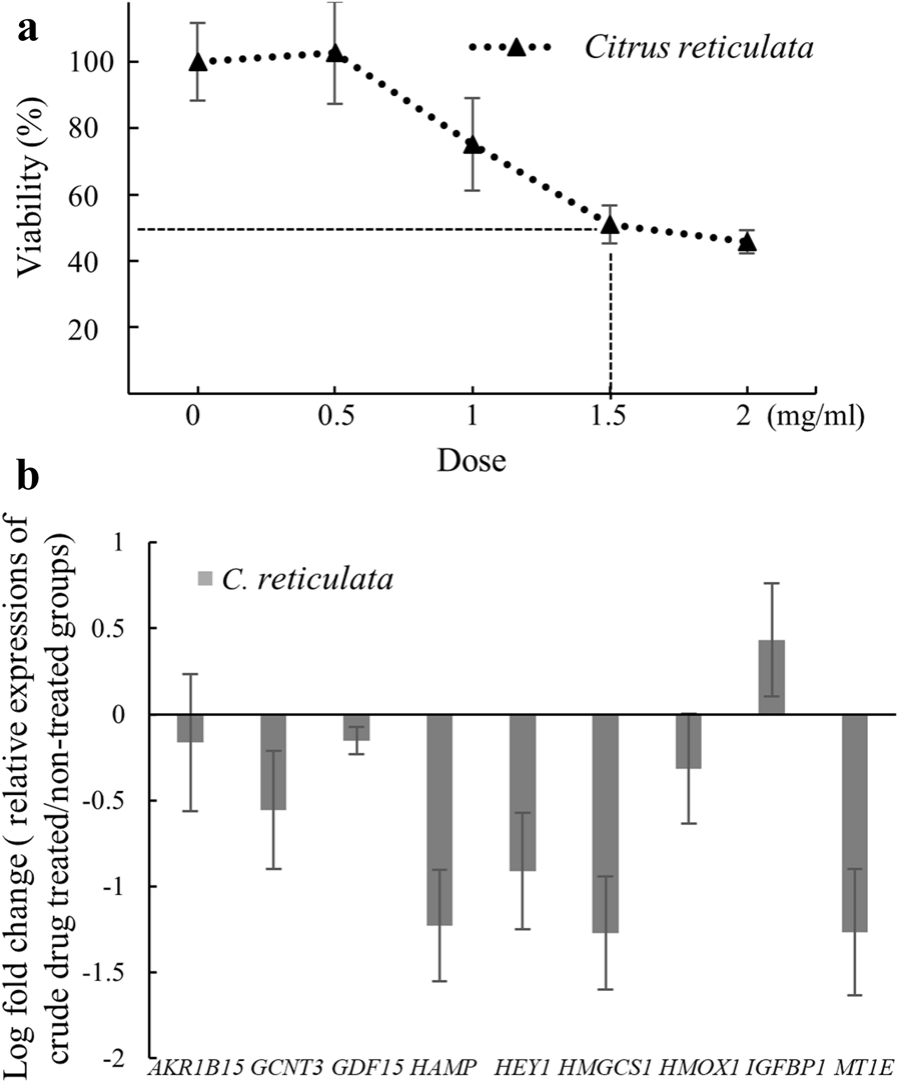
Validation of drug-related biomarkers in HepG2 cells treated with *C. reticulate*. **a** Cytotoxic effects of *C. reticulate* on HepG2 cells were measured using the MTT at 48 h. **b** Relative expression levels of drug-related genes measured in HepG2 cells treated with *C. reticulate* at a dose of 1.5 mg/mL for 24 h

To reduce false positives, we expected qi-related genes to exhibit opposite responses to treatments with qi-tonifying and qi-consuming herbs. To identify the biomarkers that can accurately reflect the qi status, we examined the expression levels of the aforementioned nine genes in the HepG2 cells treated with the peel of *C. reticulata*, a qi-consuming crude drug. HepG2 cells were treated with 1.5 mg/mL of *C. reticulata* for 24 h (Fig. 5a), and total RNA was extracted to examine expression values. As shown in Fig. 5b, *GCNT3*, *GDF15*, and *HMOX1* exhibited reciprocal responses to qi-tonifying (*A. membranaceus* and *C. pilosula*) (Fig. 4) and qi-consuming (*C. reticulata*) treatments, indicating that these three genes can be used as biomarkers to reflect the status after treatment with qi-related drugs.

### Expression patterns of *GCNT3*, *GDF15*, and *HMOX1* in two other hepatocellular cell lines

**Fig. 6:**
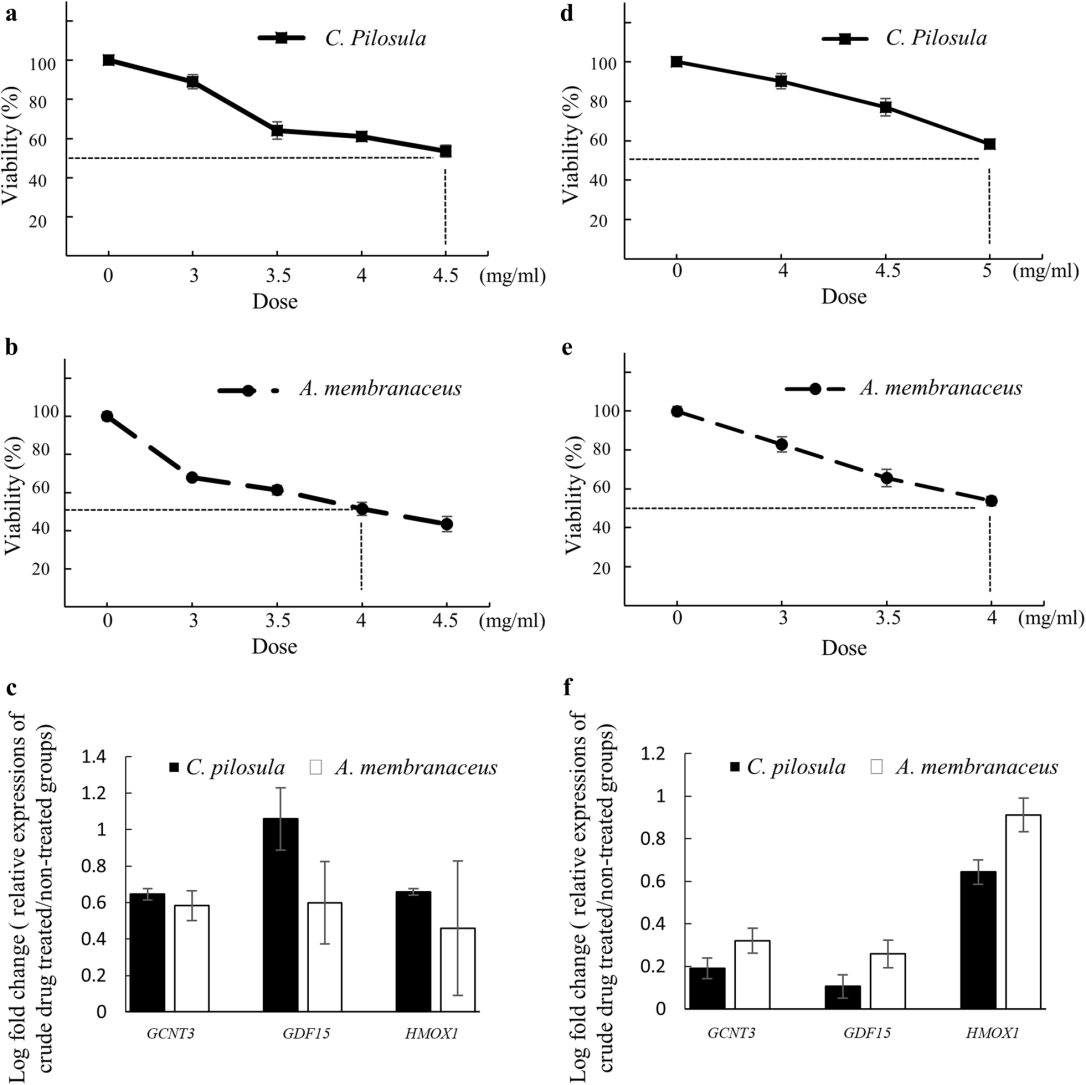
Expression levels of drug-related genes in Huh7 and L02 liver cells treated with *C. pilosula* and *A. membranaceus*. Cytotoxic effects of *C. pilosula* (**a**) and *A. membranaceus* (**b)** on Huh7 cells measured using MTT assay at 48 h. **c** Relative expression levels of drug-related genes in Huh7 cells treated with *C. pilosula* (4.5 mg/mL) or *A. membranaceus* (4 mg/mL) for 24 h. Cytotoxic effects of **d** *C. pilosula* and **e** *A. membranaceus* on L-02 cells measured by using the MTT assay at 48 h. **f** Relative expression levels of drug-related genes in L-02 cells treated with *C. pilosula* (5 mg/mL) or *A. membranaceus* (4 mg/mL) for 24 h

To exclude the cell line–specific effect, we further examined the expression of these genes in a hepatocarcinoma cell line, Huh7, and normal hepatic L-02 cells. The doses that caused 50% of growth inhibition of *C. pilosula* and *A. membranaceus* were 4.5 and 4 mg/mL in Huh7 cells respectively (Fig. 6a, b). The same experiment revealed 50% growth inhibition doses of 5 and 4 mg/mL on L-02 cells (Fig. 6d, e). Expression patterns of *GCNT3*, *GDF15*, and *HMOX1* in Huh7 and L-02 cells after *C. pilosula* or *A. membranaceus* treatments (Fig. 6c, f) were similar to those in hepatocellular carcinoma HepG2 cells (Fig. 4).

### Expression of GDF15 and HMOX1 proteins was elevated in HepG2 cells

**Fig. 7:**
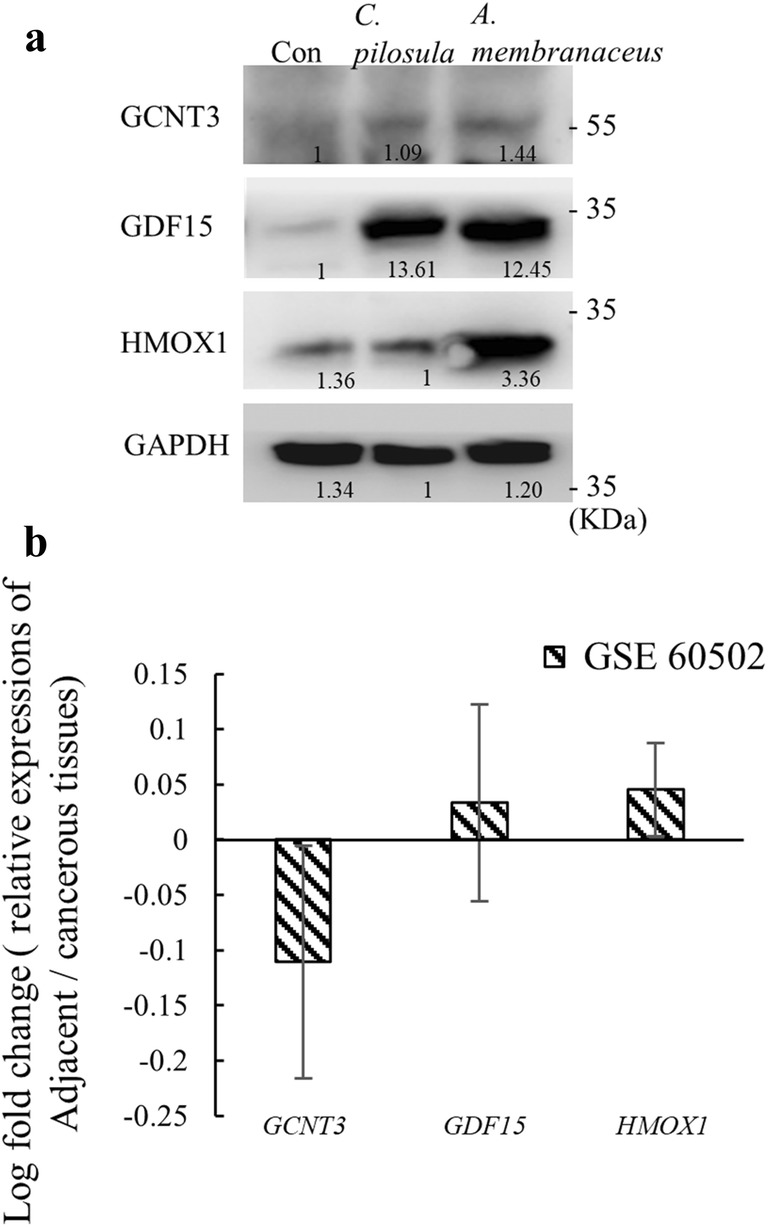
Validation of drug-related biomarkers at the protein level by using clinical data from external dataset. **a** Immunoblots of drug-related biomarker proteins. **b** Relative expression levels of drug-related genes in adjacent normal tissues compared with hepatocellular carcinoma were examined using the GEO data set, GSE60502 (https://www.ncbi.nlm.nih.gov/geo/query/acc.cgi?acc=GSE60502)

The protein expressions of GCNT3, GDF15 and HMOX1 were examined in HepG2 cells at the dose of 3 mg/mL of both *C. pilosula* or *A. membranaceus* for 48 h. Similar to the results of RNA (Fig. 6c, f), the GDF15 protein content drastically increased after treatments with *C. pilosula* or *A. membranaceus*, HMOX1 was increased after the treatment of *A. membranaceus*, and amount of GCNT3 did not change (Fig. 7a).

### Drug-related gene markers on clinical samples

After identifying the markers in the cell line model, we examined the relative expression levels of the three genes in clinical samples. The expression data were downloaded from the GEO data set GSE60502. As shown in Fig. 7b, the expression patterns of *GDF15* and *HMOX1*, but not *GCNT3*, in adjacent normal tissues compared with hepatocellular carcinoma showed patterns similar to those in cells treated with qi-tonifying drugs (Figs. 4, 6), implicating the application of these two genes (*GDF15* and *HMOX1*) as biomarkers after treatment with qi-related drugs.

## Discussion

Although the definition and characteristics of qi-related botanical drugs, such as *C. pilosula* and *A. membranaceus*, have been debated for a long time, no decisive conclusion has been reached. The key reason for the controversial results is that these drugs are prescribed exclusively on the basis of apparent symptoms, which are hard to define neither qualitatively nor quantitatively. In this study, we qualitatively defined biological effects by measuring the expression levels of related genes in hepatocellular cells and hepatocellular carcinoma samples to identify indicators that reflect the qi status after treatment with these two herbs.

*Astragalus membranaceus* and other botanic drugs are prescribed frequently in patients with liver cancer [10]. However, identifying related biomarkers for each drug was difficult because numerous variables could not be controlled in a clinical setting, such as sex, age, and dietary habits. Therefore, we used a cell model in the current study to eliminate these variables. Furthermore, genomic approaches, such as microarray technique are ideal detection methods for surveying the mechanism of botanical herbs because one herbs may contain many ingredients and may trigger many downstream pathways.

To reduce the effects of false positives, we only selected differentially expressed genes that responded to both *C. pilosula* and *A. membranaceus* (Fig. 3c). These drug-related gene candidates were mainly involved in organismal injury, cellular development, and cellular growth. These results are consistent with those in previous studies [5, 7, 8]. For example, *C. pilosula* induced proliferation and migration in RSC96 Schwann cells [18] and exhibited a hepatoprotective effect in a rat model [19]. *A. membranaceus* exhibited cardioprotective activity [20] and wound-healing effects [8]. These studies have shed some light regarding the association between qi-related drugs and biological functions and could provide clues for additional explorative experiments in the future.

After microarray analysis, nine genes with relatively high fold changes were selected for validation through qRT-PCR. Except *AKR1B15*, the remaining eight genes had expression patterns similar to those in the microarray (Fig. 4). Because the drug-related genes were expected to exhibit opposite responses when the cells were treated with qi-tonifying and qi-consuming herbs, the genes were also examined in HepG2 cells treated with the qi-consuming drug *C. reticulata*. *GCNT3*, *GDF15*, and *HMOX1* exhibited reciprocal responses to qi-tonifying (Fig. 4) and qi-consuming (Fig. 5) treatments.

The expression patterns of these three genes were examined in other hepatocellular cells. The expression patterns of *GCNT3*, *GDF15*, and *HMOX1* in Huh7 and L-02 cells after treated with qi-tonifying herbs (Fig. 6) were similar to those in hepatocellular carcinoma HepG2 cells (Fig. 4). Other hepatoma cell lines, such as Hep3B and HA22T, were not selected because both *C. pilosula* and *A. membranaceus* cannot reach fifty percent of growth inhibitory effect at the maximum dose of 5 mg/mL (data not shown). Hep3B and HA22T belong to poorly differentiated malignant hepatocellular cell lines [21] and they might show more resistance against anticancer drugs.

*Codonopsis pilosula* and *A. membranaceus* are frequently prescribed according to patients’ external symptoms. Qi deficiency is characterized by physical symptoms such as lassitude, spontaneous sweating, pale complexion, and a weak pulse, which cannot be easily quantitated. The practical significance of the experiments was to identify the gene markers for these drugs so that these drugs can be prescribed by examining the gene marker expressions in patients. Therefore, the relative expressions levels of these three drug-related genes were examined in clinical samples from public repository, Gene Expression Omnibus (GEO). We hypothesized that the expression profiles of these drug-related genes in cells treated with qi-tonifying drugs, *C. pilosula* or *A. membranaceus*, are similar to those in adjacent normal tissues compared with cancerous tissues. *GDF15* and *HMOX1*, but not *GCNT3*, were upregulated in adjacent normal tissues, which was similar to the pattern in cells treated with qi-tonifying drugs (Figs. 4, 6). Therefore, our results suggested that *GDF15* and *HMOX1* could be biomarkers for treatment with qi-related drugs. Although it remains unclear how these genes could reflect the cell status after treatment with qi-related drugs, previous studies have shown that *GDF15* promoted angiogenesis through the p53/HIF-1 alpha pathway [22], and *HMOX1* maintained bone mass by attenuating a redox imbalance [23]. However, additional experiments are required to explore the underlying mechanism by which these two genes reflect the cell status and to examine the expression of these genes in our collected clinical samples.

## Conclusions

By using the genomic approach of microarrays in hepatocellular carcinoma cells treated with qi-tonifying botanic drugs, *C. pilosula* and *A. membranaceus*, followed by qRT-PCR validation of hepatic normal cells and clinical samples, we identified drug-related genes associated with qi and their functions. Additionally, our results suggested that *GDF15* and *HMOX1* are potential biomarkers in hepatic cells, which might serve as indicators of effectiveness while prescribing these qi-related drugs.

## Additional file

**Additional file 1.** Minimum standards of reporting checklist.

## Abbreviations

TCM: traditional Chinese medicine
ITS1: internal transcribed space 1
PCA: principal components analysis
